# Platinum-induced mitochondrial OXPHOS contributes to cancer stem cell enrichment in ovarian cancer

**DOI:** 10.1186/s12967-022-03447-y

**Published:** 2022-05-31

**Authors:** Shruthi Sriramkumar, Riddhi Sood, Thomas D. Huntington, Ahmed H. Ghobashi, Truc T. Vuong, Tara X. Metcalfe, Weini Wang, Kenneth P. Nephew, Heather M. O’Hagan

**Affiliations:** 1 Cell, Molecular and Cancer Biology Graduate Program, Indiana University School of Medicine, Bloomington, IN 47405 USA; 2 Medical Sciences Program, Indiana University School of Medicine, Bloomington, IN 47405 USA; 3 Genome, Cell and Developmental Biology, Department of Biology, Indiana University Bloomington, Bloomington, IN 47405 USA; 4 Indiana University Melvin and Bren Simon Comprehensive Cancer Center, Indianapolis, IN 46202 USA; 5 Department of Anatomy, Cell Biology and Physiology, Department of Obstetrics and Gynecology, Indiana University School of Medicine, Indianapolis, IN USA; 6 Department of Medical and Molecular Genetics, Indiana University School of Medicine, Indianapolis, IN USA

**Keywords:** Ovarian cancer, Platinum, Chemoresistance, ALDH + cells, Cancer stem cells, OXPHOS, SIRT1

## Abstract

**Background:**

Platinum based agents—cisplatin and carboplatin in combination with taxanes are used for the treatment of ovarian cancer (OC) patients. However, the majority of OC patients develop recurrent, platinum resistant disease that is uniformly fatal. Platinum treatment enriches for chemoresistant aldehyde dehydrogenase (ALDH) + ovarian cancer stem cells (OCSCs), which contribute to tumor recurrence and disease relapse. Acquired platinum resistance also includes metabolic reprograming and switching to oxidative phosphorylation (OXPHOS). Chemosensitive cells rely on glycolysis while chemoresistant cells have the ability to switch between glycolysis and OXPHOS, depending on which pathway drives a selective advantage for growth and chemoresistance. High expression of genes involved in OXPHOS and high production of mitochondrial ROS are characteristics of OCSCs, suggesting that OCSCs favor OXPHOS over glycolysis. Based on connections between OCSCs, chemoresistance and OXPHOS, we hypothesize that platinum treatment induces changes in metabolism that contribute to platinum-induced enrichment of OCSCs.

**Methods:**

The effect of cisplatin on mitochondrial activity was assessed by JC1 staining and expression of OXPHOS genes by RT-qPCR. Cisplatin-induced changes in Sirtuin 1 (SIRT1) levels and activity were assessed by western blot. Small molecule inhibitors of mitochondrial complex I and SIRT1 were used to determine if their enzymatic activity contributes to the platinum-induced enrichment of OCSCs. The percentage of ALDH + OCSCs in OC cells and tumor tissue from xenograft models across different treatment conditions was analyzed using ALDEFLUOR assay and flow cytometry.

**Results:**

We demonstrate that platinum treatment increases mitochondrial activity. Combined treatment of platinum agents and OXPHOS inhibitors blocks the platinum-induced enrichment of ALDH + OCSCs in vitro and in vivo. Furthermore, platinum treatment increases SIRT1 levels and subsequent deacetylase activity, which likely contributes to the increase in platinum-induced mitochondrial activity.

**Conclusions:**

These findings on metabolic pathways altered by platinum-based chemotherapy have uncovered key targets that can be exploited therapeutically to block the platinum-induced enrichment of OCSCs, ultimately improving the survival of OC patients.

**Supplementary Information:**

The online version contains supplementary material available at 10.1186/s12967-022-03447-y.

## Background

Ovarian cancer (OC) is the 5th leading cause of cancer related deaths among women. The 5-year survival rate of OC patients has remained low for decades at 46% [1]. Surgical debulking followed by platinum and taxane-based chemotherapy are standard treatment modalities for OC patients [2]. The most common histologic sub-type of serous epithelial OC—high grade serous OC (HGSOC) initially responds well to platinum-based chemotherapy [3]. However, eventually patients with HGSOC relapse and develop a chemoresistant disease. Development of platinum resistance is the major obstacle in improving the survival of OC patients.

In the majority of solid tumors including OC, it has been firmly established that cancer stem cells (CSCs) are responsible for the development of chemoresistance and disease recurrence [4–6]. Aldehyde dehydrogenase (ALDH) is the most commonly used robust marker of CSCs [6], and ALDH + OCSCs have high tumor initiation capacity, enhanced ability to grow as spheroids and express high levels of stemness genes like *BMI1*, *OCT4* and *NOTCH3* [7, 8]. Furthermore, high expression and activity of the ALDH1A isoform strongly correlates with platinum resistant OC cells [8–10] and negatively correlates with survival of OC patients [11, 12]. Our group as well as others have demonstrated that treatment of OC cells with platinum-based agents results in enrichment of ALDH + OCSCs [7, 13, 14]. Collectively, the evidence suggests that platinum-induced enrichment of ALDH + OCSCs contributes to OC recurrence and relapse.

OCSCs are resistant to glucose deprivation and primarily rely on oxidative phosphorylation (OXPHOS) for energy requirements, which may also contribute to chemotherapy resistance [15–17]. Chemosensitive OC cells favor glycolysis while chemoresistant OC cells are able to switch between glycolysis and OXPHOS depending on which pathway provides selective advantage for growth and platinum resistance [16]. OCSCs exhibited high mitochondrial reactive oxygen species (ROS) production and high expression of enzymes involved in OXPHOS, implying that OCSCs preferentially utilize mitochondrial OXPHOS [15]. Lung and pancreatic CSCs also displayed higher membrane potential and lower glucose consumption rates compared to non-CSCs [18, 19]. Thus, there is strong evidence supporting altered metabolism as an important factor driving platinum resistance.

ALDH is a nicotinamide adenine dinucleotide (NAD +) dependent enzyme [20, 21]. We and others have demonstrated that platinum treatment results in an increase in expression of nicotinamide phosphoribosyltransferase (NAMPT)—a rate limiting enzyme in NAD + biosynthesis salvage pathway. The subsequent increase in cellular NAD + levels promotes ALDH + OCSC enrichment and contributes to platinum resistance [13, 14]. However, it is well-established that platinum resistance is a multifactorial phenomenon [22] and it is likely that additional metabolic pathways contribute to the enrichment of ALDH + OCSCs and chemoresistance.

In the present study, we examined the effect of platinum treatment of HGSOC cells on mitochondrial activity. We observed that mitochondrial membrane potential (∆Ψ_M_) and expression of genes involved in mitochondrial OXPHOS increased 16 h after platinum treatment. Concomitantly, expression of genes in the glycolysis pathway was decreased by platinum treatment. Treatment of OC cells with OXPHOS inhibitors blocked the platinum-induced enrichment of ALDH + OCSCs, and increased deacetylase activity of sirtuin 1 (SIRT1) was required for platinum-induced enrichment of ALDH + OCSCs. In addition, we observed that SIRT1-regulated expression of mitochondrial transcription factor A (TFAM) likely contributed to the platinum mediated increase in mitochondrial activity. This first report demonstrating that OXPHOS inhibitors block platinum-induced enrichment of OCSCs supports further investigation into the role of mitochondrial OXPHOS in ovarian tumor recurrence.

## Methods

### Cell culture

HGSOC cell lines used in the study were maintained at 37 °C and 5% CO_2_ as described previously [23]. OVCAR5, OVCAR3 and OVSAHO cell lines were authenticated by ATCC in 2018. OVCAR5, OVCAR3 cells were cultured in DMEM 1X (Corning, #MT10013CV) and OVSAHO were cultured RPMI 1640 (Corning, #MT10040CV), containing 10% FBS (Gibco, #16000044) without antibiotics. All the cell lines used in the study were passaged less than 15 times. A 1.67 mM stock solution of cisplatin (Millipore Sigma, #232120) was made using 154 mM NaCl (Macron Fine chemicals, #7581-12) in water. All the HGSOC cell lines used in the study were treated with respective IC50 doses of cisplatin determined previously by Haley et al. using a 24 h treatment followed by a 72 h recovery period (OVCAR5 12 µM; OVSAHO 4 µM; OVCAR3 15 µM) [24]. Stock solutions of Rotenone (Sigma, #R8875-1G; 100 mM), IACS—010759—OXPHOS Inhibitor (MedChemExpress, #HY-112037; 10 mM), oligomycin (SelleckChem, #S1478; 10 mM), and SIRT1 inhibitor Ex-527 (Sigma, E7034; 10 mM) were made in DMSO. For all the experiments using these inhibitors, an equivalent amount of DMSO or inhibitors were added along with cisplatin and cells were incubated for 16 h at 37 °C and 5% CO_2_. All the treatment doses are specified in the figure legends.

### JC-1 staining

OVCAR5 (1 × 10^6^) and OVSAHO cells (1.5 × 10^6^) were cultured in 100 mM plates for 24 h and treated with respective IC_50_ doses of cisplatin (OVCAR5 12 µM; OVSAHO 4 µM [24]) for 16 h. JC-1 staining was performed as per the manufacturer’s protocol (Thermo Fisher, #T3168). Briefly, cells were collected and washed with PBS and then resuspended in 1 ml PBS. Stock solutions of JC-1 were made in DMSO at a concentration of 5 mg/ml. Then JC-1 stain was added to cells at a final concentration of 2 µg/ml and cells were incubated at 37 °C, 5% CO_2_ for 30 min. Following incubation, the cells were filtered through 30 µm filters and analyzed by flow cytometry. For all the JC-staining experiments a no stain sample was used for compensation control. Analysis of JC-1 staining was performed using Flow Jo software (Becton, Dickinson & Company, RRID: SCR_008520). The mean red and green intensity of all cells in each biological replicate was determined using Flow Jo software. Then the ratio of red to green intensity was calculated for each biological replicate and the mean of ratio of red to green intensities of all 3 biological replicates was plotted in the graphs.

### RNA isolation and reverse transcriptase—quantitative PCR (RT-qPCR)

Isolation of total RNA from cell pellets was performed using RNAeasy mini kit (Qiagen, #74104) as per the manufacturer’s protocol. Maxima first strand cDNA synthesis kit (Thermo Fisher, #K1642) for quantitative reverse transcription PCR was used to synthesize cDNA. FastStart Essential DNA green master (Roche, #06402712001) was used to perform RT-qPCR. Primers sequences of all candidate genes and their respective efficiencies are in Additional file 2: Table S1. Relative gene expression was calculated using the Pfaffl method to account for differences in primer efficiencies between the primers for the genes of interest and the housekeeping gene *Actin B*.

### ALDEFLUOR assay

OVCAR5 (1.0 × 10^5^) and OVSAHO (1.5 × 10^6^) were cultured in 100 mm dishes. Approximately 24 h after plating, cells were treated with cisplatin alone or in combination with DMSO or appropriate inhibitors and incubated at 37 °C, 5% CO_2_ for 16 h. After the incubation time ALDEFLUOR assay (Stem Cell Technologies, #01700) was performed as described previously [14].

### Flow cytometry

LSR II Flow cytometer (BD Biosciences) was used for the analysis of both the JC-1 and ALDEFLUOR assay. JC1 J-monomers and J-aggregates was measured using 488 nm excitation and signal was detected using AF488 (green) and PE-A (red), respectively. Samples with no stain were analyzed for every experiment for compensation control. For all the ALDEFLUOR assays, 488 nm excitation was used, and the signal was detected using 530/30 filter to measure ALDH activity. To determine the percentage of ALDH positive cells in different conditions, the sample specific DEAB negative control was used. All the data analysis was done using FlowJo software (Becton, Dickinson & Company).

### Generation of stable knockdown lines

For SIRT1 knockdown (Sigma, NM_012238, TRCN0000018981, TRCN0000018983) and empty vector (EV) TRC1, the lentiviral shRNA knockdown protocol from the RNAi consortium of the Broad Institute was used as described previously [23]. Briefly, OVCAR5 cells were transduced with appropriate amount of concentrated virus after titration along with 8 μg/ml polybrene. The following day media was changed and media containing 2.5 μg/ml puromycin was added for 3 days to select for infected cells. Approximately 3 days later cells were split and used for experiments.

### Mitochondrial DNA (mtDNA) content analysis

1 × 10^6^ OVCAR5 cells were plated and incubated at 37 °C and 5% CO_2_ for approximately 24 h. Then cells were treated with the IC50 dose of cisplatin (12 μM for OVCAR5). After 16 h treatment, cells were collected, DNA was isolated using DNAeasy blood and tissue kit (Qiagen, 69504) and quantified using a nanodrop. Mitochondrial DNA content relative to a nuclear DNA was quantified by qPCR and the Delta Delta Ct using primers that amplify mitochondrial DNA regions (NADH dehydrogenase sub-unit 1 and NADH dehydrogenase sub-unit 5) and a nuclear DNA (nDNA) region (β_2_—macroglobulin) as described previously [25–27]. See Additional file 2: Table S2 for primers.

### Xenograft studies

All the animal studies were performed in accordance with the Association for Assessment and Accreditation of Laboratory Animal Care International and approved by Indiana University Bloomington Institutional Animal Care and Use Committee as described previously [14]. A power analysis performed using data from a pilot experiment indicated that 4 mice per group would provide 80% power to detect a difference in %ALDH + cells at p < 0.05. 2 × 10^6^ OVCAR3 cells were injected subcutaneously in the flanks of NSG mice (IUSCCC In Vivo Therapeutics Core). Once the established tumors reached > 100 mm^3^, they were randomized into 3 different treatment groups and treated with vehicle alone or combination of carboplatin and vehicle or IACS-010759 (OXPHOS inhibitor). Carboplatin was administered once a week intraperitoneally at 50 mg/kg for 3 weeks. IACS-010759 was prepared in 10% DMSO and 90% corn oil. IACS-010759 was administered orally at 7.5 mg/kg 5 days a week for 3 weeks. At the end of the treatment schedule, mice were sacrificed and tumors were collected, disassociated and used for ALDEFLUOR assay as we have done previously [7, 14]. Tumor dissociation kit and gentle MACS dissociator (Miltenyi Biotech) was used to dissociate the tumors into single cells as per manufacturer’s protocol.

### Correlation analysis using cBioportal

Correlation analysis of expression of *SIRT1* and *TFAM* was performed using the cBioportal [28, 29] and RNA-seq data of ovarian cancer samples from The Cancer Genome Atlas [30].

### Spheroid formation assay

1.5 × 10^3^ cells pre-treated with DMSO or IACS alone or in combination with cisplatin (cisplatin, 6 μM; IACS—010,759, 1 μM) for 3 h were plated in 24-well low attachment plates (Corning, 3473) in stem cell media [DMEM-F12 (Corning, #10-017-CV) with 100 U Penicillin–Streptomycin, 0.4% BSA, 10 ng/ml bFGF (Invitrogen, #13256-029), 20 ng/ml EGF (Biolegend, 585,506), 5 μg Insulin (Sigma, 19278)]. The plates were incubated for 14 days at 37 °C, 5% CO_2_. Fresh stem cell media was supplemented every 3 days and the spheroids were imaged at 14 days. The percent area of spheroids in each well was calculated using ImageJ for each biological replicate and the graph was plotted using all the three replicates.

### Antibodies

For western blot, the following antibodies were used: anti-pATMS1981 (Cell Signaling Technology (CST), #13050, 1:1000), anti-AcH4K16 (CST, #13534, 1:1000), anti-total H2A (CST, #12349, 1:1000), anti-SIRT1 (Santa Cruz, sc-74465, 1:1000), anti-HIF1a (CST, #36169,1:1000), anti-Myc (Abcam, ab32072,1:1000), anti-actin B (CST, #4970) and anti-vimentin (CST, #5741).

### Statistics

The percentage of ALDH + cells across different conditions after ALDEFLUOR assay and percent area of spheroid assay was evaluated by one-way ANOVA with multiple comparisons using Graphpad prism and data is presented as ± SEM. Data of JC-1 staining, RT-qPCR and densitometry were evaluated by Student *t* test in Graphpad prism and excel.

## Results

### Platinum treatment increases mitochondrial OXPHOS activity

**Fig. 1:**
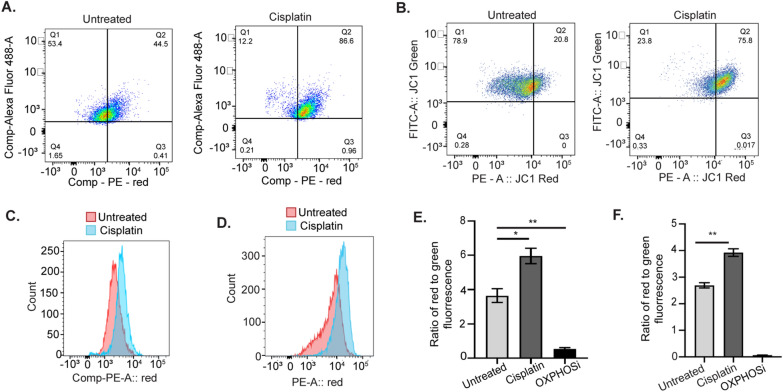
Mitochondrial OXPHOS activity increases in response to cisplatin treatment in OC cells. Scatter plots of OVCAR5 (**A**) and OVSAHO (**B**) cells untreated or treated with 12 µM and 4 µM cisplatin, respectively, for 16 h followed by staining with JC-1 for 30 min and analysis by flow cytometry. Histogram of untreated and cisplatin treated OVCAR5 (**C**) and OVSAHO (**D**) cells after JC-1 staining as in **A** and **B**. Ratio of red to green fluorescence intensity in untreated and cisplatin treated OVCAR5 (**E**) and OVSAHO (**F**) cells after JC-1 staining as in **A** and **B**. The OXPHOS inhibitor carbonyl cyanide 3-chlorophenylhydrazone (OXPHOSi) was used as an assay control. Graphs display mean ratio of red to green fluorescence ± SEM (N = 3). Student t-test was used to calculate statistical significance. For all untreated versus cisplatin treated, *P* values *< 0.05, **< 0.005 and ***< 0.0005

Development of platinum resistance in OC has been linked to increased reliance of cancer cells on OXPHOS [31]. Therefore, first we determined if mitochondrial membrane potential (∆Ψ_M_) [32] increased after treatment of OC cells with cisplatin. To measure ∆Ψ_M_, a lipophilic, cationic dye called JC-1 was used. JC-1 enters the mitochondria of healthy cells with normal ∆Ψ_M_ and spontaneously aggregates as red fluorescent J-aggregates; in contrast, in cells with disrupted ∆Ψ_M_, JC-1 will not form aggregates and retains its green fluorescence [32]. Treatment of HGSOC cells—OVCAR5, OVSAHO with their respective IC_50_ doses of cisplatin for 16 h followed by JC-1 staining resulted in a shift in the population of cells from low red fluorescence to high red fluorescence (Fig. 1A, B, Additional file 1: Figure S1A), indicating an increase in mitochondrial J-aggregates after cisplatin treatment. A histogram of the red fluorescence for JC-1 staining in untreated and cisplatin treated OVCAR5 and OVSAHO cells confirmed that cisplatin treated cells shifted towards higher red fluorescence (Fig. 1C, D). Furthermore, the ratio of red to green fluorescence significantly increased after cisplatin treatment of both OVCAR5 and OVSAHO cells (Fig. 1E, F). Carbonyl cyanide m-chlorophenyl hydrazone (CCCP—OXPHOSi), which is known to reduce ∆Ψ_M_ [33], was used as an assay control and reduced the ratio of red to green fluorescence as expected (Fig. 1E, F). Treatment of OVCAR5 cells with cisplatin for 16 h had no effect on the mitochondrial DNA (mtDNA) content relative to nuclear DNA content (Additional file 1: Figure S1B), further supporting that the cisplatin-induced increase in OXPHOS was due to an increase in mitochondrial activity, not an increase in mitochondrial number.

### Cisplatin treatment induces OXPHOS gene expression

**Fig. 2:**
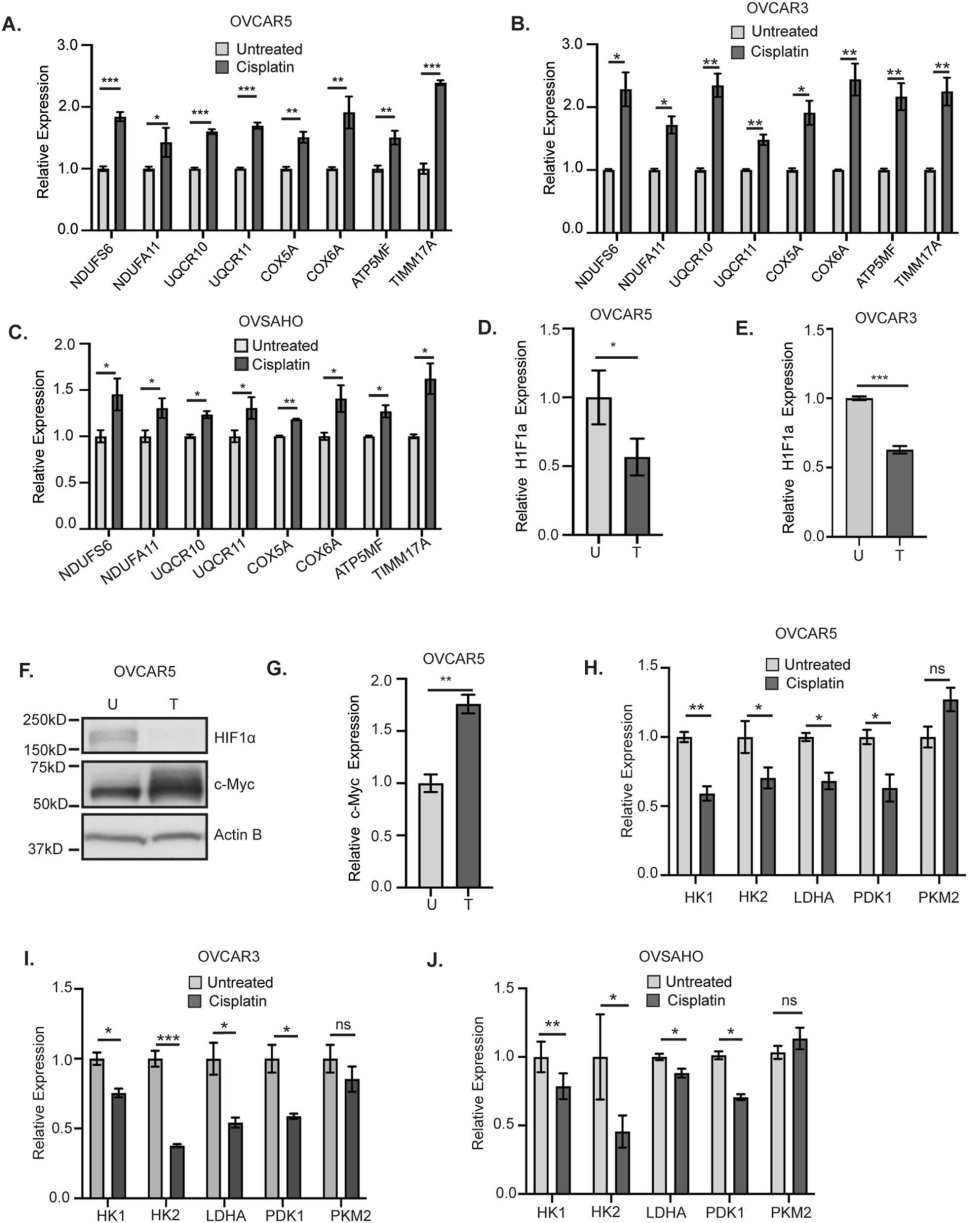
Expression of genes involved in mitochondrial OXPHOS increases after cisplatin treatment. Gene expression of the indicated genes by RT-qPCR in OVCAR5 (**A**) OVCAR3 (**B**) and OVSAHO (**C**) cells untreated or treated with 12 µM, 15 µM or 4 µM cisplatin, respectively, for 16 h. Expression of *HIF-1α* in OVCAR5 (**D**) and OVCAR3 (**E**) cells treated as in **A** and **C**. **F** OVCAR5 cells were untreated (U) or treated with 12 µM cisplatin for 16 h (T). Cell lysates were collected and analyzed for indicated proteins by western blot. **G** c-Myc expression in untreated (U) or 12 µM cisplatin treated (T) OVCAR5 cells. Expression of the indicated glycolysis genes in OVCAR5 (**H**), OVCAR3 (**I**) and OVSAHO cells (**J**) treated as in **A**, **B** and **C**, respectively. Graphs display mean fold change ± SEM relative to untreated. Expression of all the genes was normalized to the house keeping gene *Actin B*. For all untreated versus cisplatin treated, *P* values *< 0.05, **< 0.005 and ***< 0.0005

To determine if cisplatin treatment increased expression of genes of mitochondrial OXPHOS complexes, we performed RT-qPCR of untreated and 16 h cisplatin-treated cells. Increased expression of Complex I subunits—*NADH ubiquinone oxidoreductase subunits S6, A11 *(*NDUFS6*, *NDUFA11*); Complex III subunits—*ubiquinol-cytochrome C reductase subunit X*, *XI *(*UQCR10*, *UQCR11*); Cytochrome C oxidase complex subunits 5A, 6A (*COX5A*, *COX6A*); ATP synthase membrane subunit F (*ATP5MF*) and *translocase of the inner mitochondrial membrane *(*TIMM17A*) was observed in OVCAR5, OVCAR3, and OVSAHO cells after cisplatin treatment (Fig. 2A–C). Hypoxia-inducible factor 1 (HIF-1) is known to inhibit mitochondrial biogenesis and respiration by negatively regulating the activity of transcription factor c-Myc [34, 35]. Treatment of OVCAR5 and OVCAR3 cells with cisplatin for 16 h reduced the expression and protein levels of HIF1α (Fig. 2D–F, Additional file 1: Figure S2A). Additionally, cisplatin treatment increased *c-Myc* expression and protein levels in OVCAR5 cells (Fig. 2F, G) but not in OVCAR3 cells (Additional file 1: Figure S2A, B). HIF-1 regulates the expression of glycolytic genes like *lactate dehydrogenase A *(*LDHA*), *Pyruvate dehydrogenase kinase *(*PDK1*) and *Pyruvate kinase isozyme M2 *(*PKM2*) [36, 37]. We also examined the expression of hexokinase genes *hexokinase 1* (*HK1*) *and hexokinase 2* (*HK2*), which are involved in the first step in glycolysis [38]. Treatment of OVCAR5, OVCAR3, and OVSAHO cells with cisplatin for 16 h decreased expression of *HK1, HK2, LDHA,* and *PDK1* but not *PKM2* (Fig. 2H–J). Altogether, our findings suggest that platinum treatment increased ∆Ψ_M_, increased expression of genes related to OXPHOS and decreased expression of genes involved in glycolysis.

### OXPHOS is essential for the platinum-induced enrichment of ALDH + cells

**Fig. 3:**
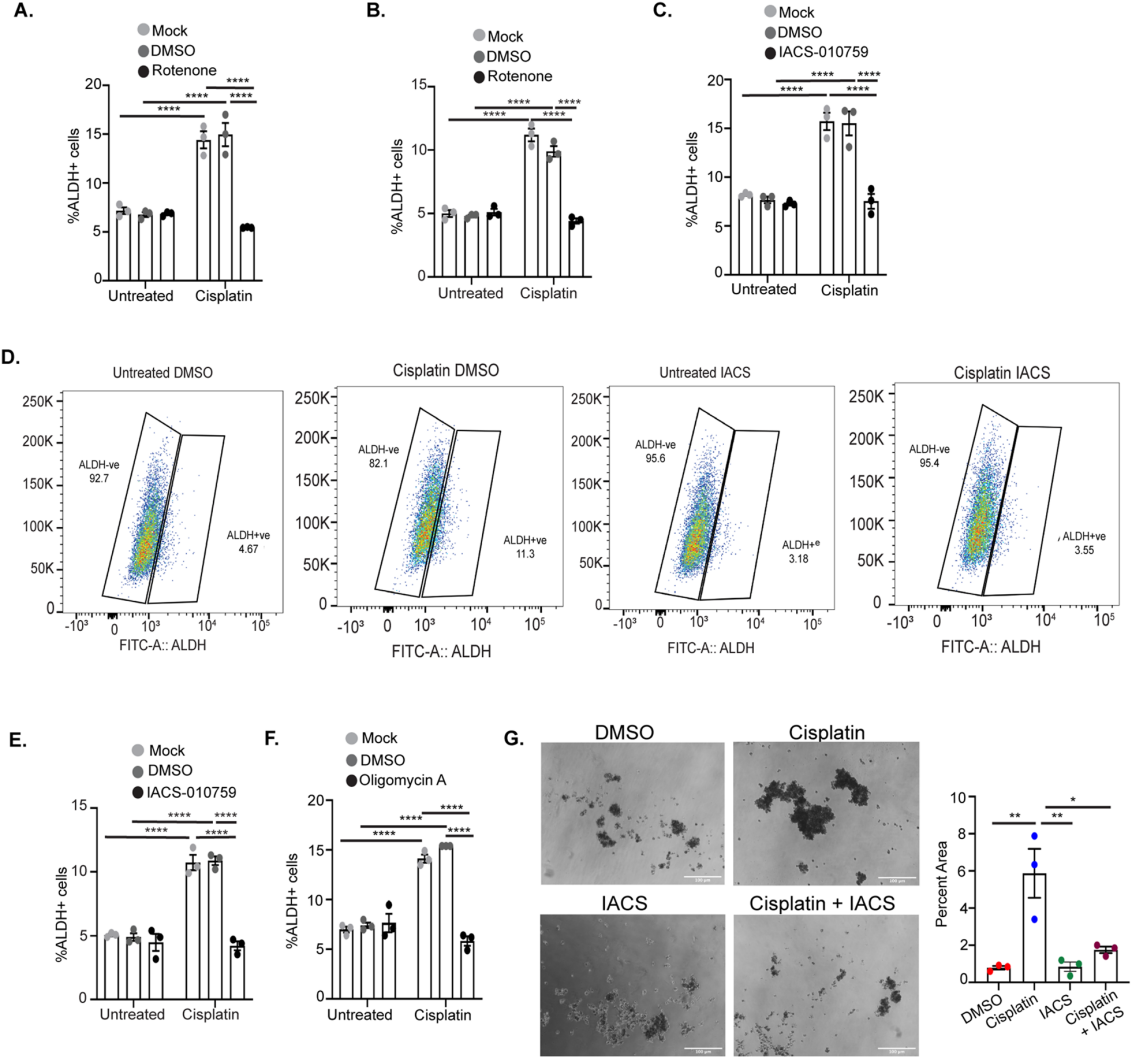
Mitochondrial OXPHOS inhibitors in combination with cisplatin block the platinum-induced increase in percent ALDH + cells. Percent ALDH + OVCAR5 (**A**) and OVSAHO cells (**B**) untreated or treated with 12 µM or 4 µM cisplatin, respectively, alone or in combination with DMSO or 5 µM Rotenone for 16 h followed by ALDEFLUOR assay. Percentage ALDH + OVCAR5 (**C**) and OVSAHO (**D**, **E**) cells treated with cisplatin alone or in combination with DMSO or 1 µM IACS-010759 for 16 h followed by ALDEFLUOR assay. **D** Shows gates used to determine ALDH + cells for one biological replicate of OVSAHO cells treated with DMSO or IACS in combination with cisplatin. **F** Percent ALDH + OVCAR5 cells treated with cisplatin alone or in combination with DMSO or 1 µM oligomycin for 16 h followed by ALDEFLUOR assay. Graphs display mean ± SEM percent ALDH + cells in N = 3 biological replicates. **G** Spheroid formation assay in OVCAR5 cells pre-treated for 3 h with DMSO or 1 μM IACS-010759 alone or in combination with 6 μM cisplatin and cultured for 14 days. For all untreated versus cisplatin treated, *P* values *< 0.05, **< 0.005, ***< 0.0005, ****< 0.0001

Platinum treatment has been previously demonstrated to enrich the ALDH + OCSC population [7, 13, 14]. To determine if the increase in OXPHOS in response to cisplatin treatment is essential for the platinum-induced enrichment of ALDH + cells, we treated OVCAR5 and OVSAHO cells with cisplatin alone or in combination with 5 µM Rotenone, a mitochondrial complex I inhibitor, or DMSO and performed the ALDEFLUOR assay. As expected, treatment of OVCAR5 and OVSAHO cells with cisplatin alone or in combination with DMSO increased the percentage of ALDH + OCSCs (Fig. 3A, B). Rotenone treatment blocked the platinum-induced increase in %ALDH + OCSCs without changing the basal percentage of ALDH + cells. IACS—010759 is a novel small molecule OXPHOS inhibitor, which like rotenone also inhibits complex I of the mitochondrial respiratory electron transport chain [39]. Treatment of OVCAR5 and OVSAHO cells with 1 µM IACS-010759 in combination with cisplatin abrogated the platinum-induced enrichment of ALDH + cells (Fig. 3C–E; Additional file 1: Figure S3A–C). IACS-010759 treatment did not affect the platinum-induced change in the expression of OXPHOS genes, *c-Myc* or *HIF1α* in OVCAR5 cells (Additional file 1: Figure S4A, B). Oligomycin inhibits the ATP synthase complex in the mitochondrial respiratory electron transport chain [40], and combination treatment of 1 µM oligomycin plus cisplatin blocked the platinum-induced increase in ALDH + cells (Fig. 3F). Several studies have established that cancer stem cells have the ability to grow in anchorage independent conditions forming spheroids [41, 42]. As previously demonstrated by our group, cisplatin treatment of OVCAR5 cells increased their ability to form spheroids under anchorage independent conditions compared to DMSO treated controls (Fig. 3G) [7]. Furthermore, treatment of OVCAR5 cells with IACS-010759 in combination with cisplatin blocked the cisplatin-induced increase in spheroid formation (Fig. 3G). Altogether, these data suggest that the increase in mitochondrial activity observed after cisplatin treatment is required for the platinum-induced enrichment of ALDH + OCSCs.

### SIRT1 contributes to the platinum-induced enrichment of ALDH + cells

**Fig. 4:**
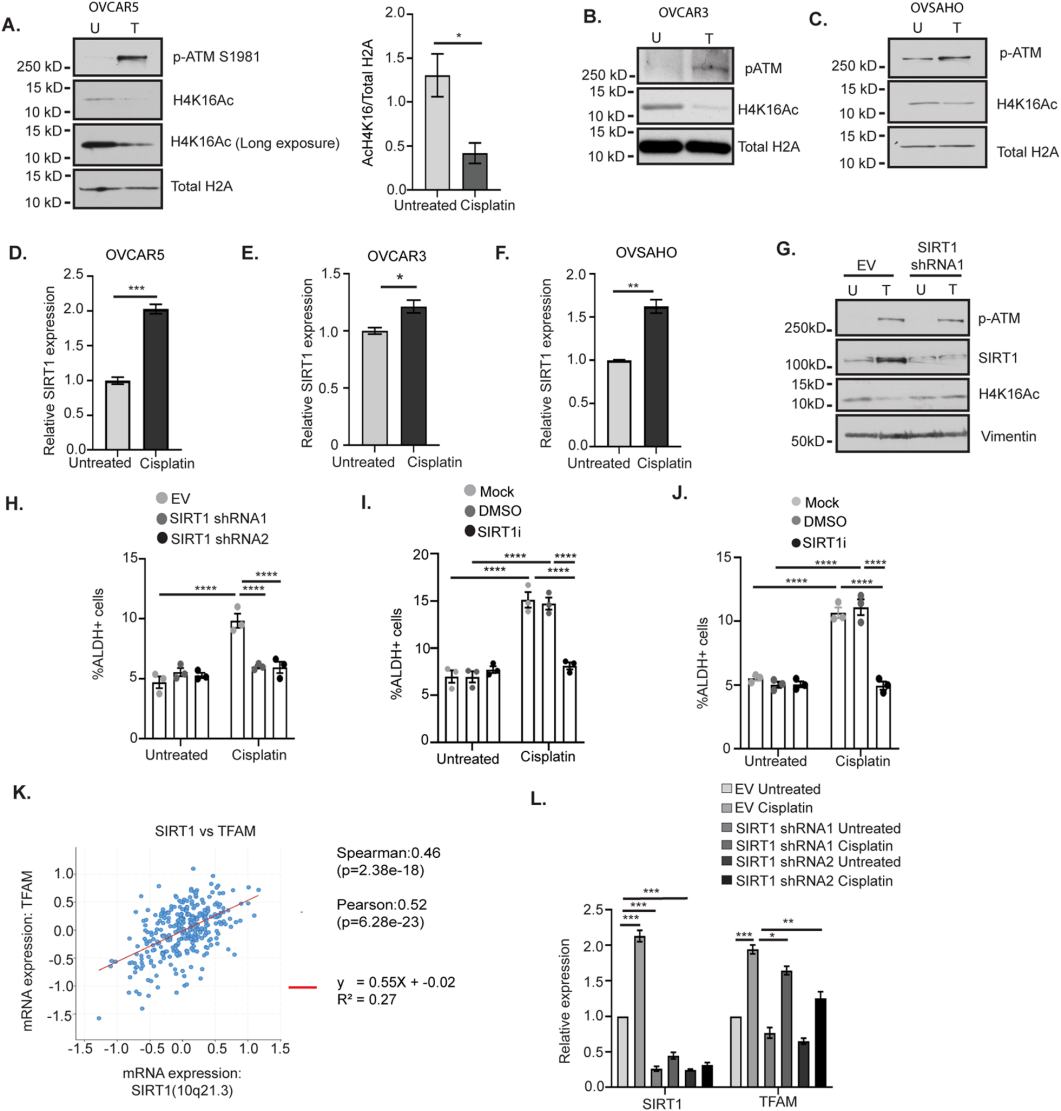
Platinum-induced increase in SIRT1 activity contributes to the enrichment of ALDH + cells. OVCAR5 (**A**), OVCAR3 (**B**) and OVSAHO (**C**) were untreated (U) or treated with respective IC50 dose of cisplatin (T) for 16 h (OVCAR5—12 μM, OVCAR3—15 μM and OVSAHO—4 μM). Cell lysates were collected and analyzed by western blot. **A** Graph displays mean ± SEM densitometric analysis of N = 3 biological replicates of H4K16ac relative to total H2A. Relative expression of SIRT1 in OVCAR5 (**D**), OVCAR3 (**E**) and OVSAHO (**F**) cells treated as in **A**, **B** and **C**, respectively. **G** OVCAR5 cells infected with empty vector (EV) or SIRT1 viral shRNA followed by treatment as in **A**. Lysates were collected and analyzed by western blot for the indicated proteins. **H** OVCAR5 cells infected EV or SIRT1 viral shRNA, untreated or treated as in A followed by ALDEFLUOR assay. Graph shows mean ± SEM percent ALDH + cells in N = 3 biological replicates. Percent ALDH + OVCAR5 (**I**) and OVSAHO (**J**) cells treated with cisplatin alone or in combination with DMSO or 3 µM Ex-527 for 16 h followed by ALDEFLUOR assay. Graph depicts mean ± SEM percent ALDH + cells in N = 3 biological replicates. (**K**) Correlation analysis of SIRT1 and TFAM expression using RNA-seq data from TCGA OC patient dataset. **L** Relative expression of SIRT1 and TFAM in OVCAR5 cells infected with EV or SIRT1 viral shRNA and treated as in **A**. Graph displays mean relative expression ± SEM to the untreated. Gene expression was normalized to the house keeping gene *Actin B*. For all comparisons, *P*—*< 0.05, **< 0.005, ***< 0.0005, ****< 0.0001

SIRT1, a NAD + -dependent deacetylase of acetylated histone 4 lysine 16 (H4K16Ac) and non-histone proteins, has been implicated in the regulation of metabolic processes [43, 44]. Cellular NAD + levels increase and regulate the deacetylase activity of SIRT1 after platinum treatment [13, 14, 45]. Therefore, we hypothesized that SIRT1 activity contributes to the observed cisplatin-induced metabolic changes that result in ALDH + OCSC enrichment. Cisplatin treatment for 16 h reduced the levels of H4K16Ac relative to untreated in OVCAR5, OVCAR3 and OVSAHO cells (Fig. 4A–C). Phosphorylation of ATM (p-ATM) at serine 1981 was used as a control for platinum-induced DNA damage response and we observed an expected increase in pATM after platinum treatment in all cell lines (Fig. 4A–C). Treatment of OVCAR5, OVCAR3, and OVSAHO cells with the respective IC50 doses of cisplatin for 16 h also resulted in increased SIRT1 mRNA levels (Fig. 4D–F) and increased SIRT1 protein levels in OVCAR5 cells (Fig. 4G). Furthermore, SIRT1 knockdown (KD) using shRNA rescued the platinum-induced decrease in H4K16Ac (Fig. 4G), indicating that SIRT1 likely deacetylates H4K16 in response to cisplatin treatment.

Next, to determine if SIRT1 plays a role in the platinum-induced enrichment of OCSCs, we combined SIRT1 KD or inhibition with platinum treatment. SIRT1 KD using two different shRNAs in OVCAR5 cells followed by cisplatin treatment blocked the platinum-induced increase in ALDH + cells without altering the baseline percentage of ALDH + cells (Fig. 4H). Additionally, inhibiting SIRT1 by treating cells with the SIRT1 inhibitor Ex-527 (3 µM) abrogated the platinum-induced increase in ALDH + cells in both OVCAR5 and OVSAHO cells (Fig. 4I, J).

SIRT1 has been shown to promote OXPHOS and mitochondrial biogenesis in adipocytes and hepatocellular carcinoma cells by regulating the expression of mitochondrial transcription factor A (TFAM) [46–48]. TFAM is important for proper transcription of mitochondrial and OXPHOS genes [49, 50]. To test the hypothesis that SIRT1 promoted the platinum-induced increase in ALDH + cells by promoting the increase in mitochondrial OXPHOS, we first analyzed the correlation between SIRT1 and TFAM expression in publicly available RNA-sequencing datasets from OC patients [30]. SIRT1 expression was positively correlated with TFAM expression (Fig. 4K). Additionally, treatment of OVCAR5 and OVCAR3 cells with cisplatin increased expression of TFAM (Fig. 4L, Additional file 1: Figure S4C). Next, we determined if the expression of TFAM is dependent on SIRT1 in response to cisplatin treatment in OC cells. SIRT1 KD using two different shRNAs resulted in a modest but significant reduction in the platinum-induced increase in TFAM expression (Fig. 4L). Altogether, these data suggest that SIRT1 activity plays a key role in platinum-induced enrichment of ALDH + cells in OC.

### OXPHOS inhibition blocks the platinum-induced enrichment of ALDH + cells in vivo

**Fig. 5:**
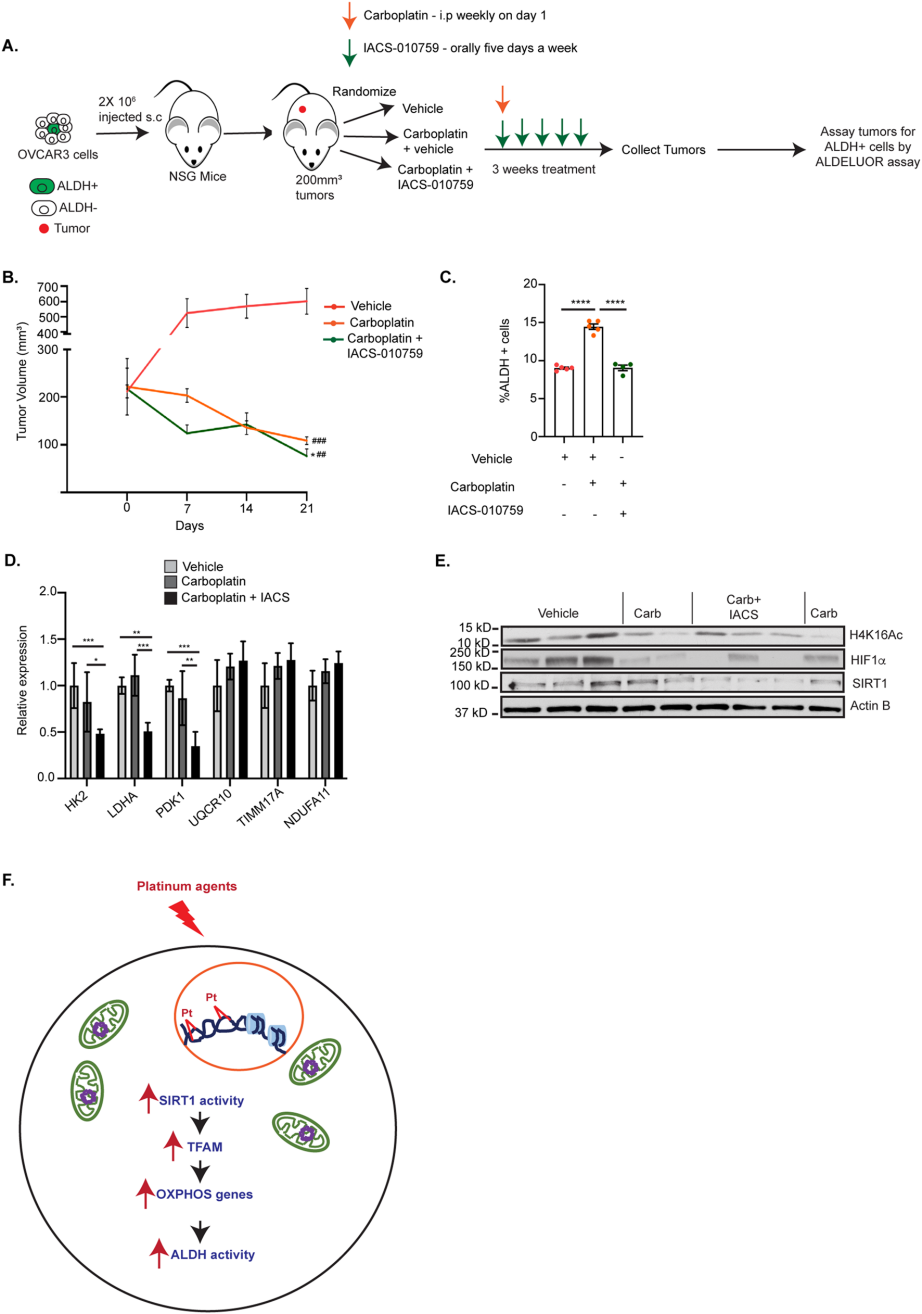
OXPHOS inhibition blocks the platinum-induced enrichment of ALDH + cells in vivo. **A** 2 × 10^6^ OVCAR3 cells were injected s.c in 6–7 weeks old NSG mice. Once the tumors were  > 100 mm^3^, mice were randomized into three groups and treated with vehicle alone or combination of vehicle + carboplatin or IACS-010759 for 3 weeks as indicated. At the end of the study, tumors were collected, dissociated into single cells and the ALDEFLUOR assay was performed. **B** Tumor volumes were measured using a digital caliper through 3 weeks of treatment by the same investigator throughout the study. N = 4–5 mice per group. *P relative to carboplatin + vehicle. ^#^*P* relative to vehicle. **C** Percentage of ALDH + cells in dissociated xenograft tumor samples using ALDEFLUOR assay. **D** Expression of glycolysis and OXPHOS genes in tumor xenografts. Graph indicates mean ± SEM in the different treatment groups. N = 4–5 mice per group. For all treated with vehicle alone versus carboplatin + vehicle or IACS-010759, *P* values *< 0.05, **< 0.005, ***< 0.0005, ****< 0.0001. **E** Western blot of the indicated proteins in whole cell lysates from tumor xenografts. **F** Model for how platinum treatment of HGSOC cells results in increased mitochondrial activity that contributes to the platinum-induced enrichment of ALDH + OCSCs. Platinum induces an increase in SIRT1 activity that increases TFAM levels, which in turn likely increases expression of OXPHOS genes that leads to an increase in mitochondrial activity

We and others have demonstrated that platinum treatment results in enrichment of ALDH + OCSCs in vitro and in vivo [7, 13, 51, 52]. Furthermore, we demonstrated that treatment of OC cells with OXPHOS inhibitors in combination with cisplatin blocked the platinum-induced enrichment of ALDH + cells in vitro without affecting the basal ALDH percentage (Fig. 3A–F). Therefore, it was of interest to determine if OXPHOS inhibition blocks the platinum mediated increase in ALDH + OCSCs in vivo in mouse xenografts. As shown in Fig. 5A, 2 million OVCAR3 cells were injected subcutaneously in the flanks of NSG mice and after establishment of tumors, randomized mice received vehicle alone or a combination of carboplatin + vehicle or OXPHOS inhibitor IACS-010759. As expected, platinum treatment significantly reduced the tumor volume compared to the vehicle only group (Fig. 5B). In the group treated with carboplatin + IACS-010759, tumor volume was less than the group treated with carboplatin alone at the end of the study (Fig. 5B). We observed the expected increase in the percentage of ALDH + cells in the carboplatin + vehicle compared to the vehicle only group (Fig. 5C), consistent with previous studies by us and others [7, 13, 51, 52]. The combination treatment of IACS-010759 + carboplatin abrogated the platinum-induced enrichment of ALDH + cells in vivo (Fig. 5C). Somewhat unexpectedly, the expression of glycolysis genes *HK2*, *LDHA* and *PDK1* decreased in tumors from mice treated with carboplatin in combination with IACS whereas the expression of OXPHOS genes did not change in any treatment group (Fig. 5D). SIRT1 protein levels in whole cell lysates from tumor xenografts were variable across treatment groups (Fig. 5E). However, consistent with our in vitro data, H4K16Ac levels were decreased in xenografts from mice treated with carboplatin alone or in combination with IACS compared to vehicle alone suggesting increased SIRT1 activity following carboplatin treatment (Fig. 5E). Tumors from carboplatin and carboplatin plus IACS treated mice also had reduced levels of HIF1α compared to vehicle alone (Fig. 5E). However, the reduction in tumor size in carboplatin treated groups make this result difficult to interpret. Overall, our data suggests that inhibiting OXPHOS using the complex I mitochondrial inhibitor IACS during carboplatin treatment blocks platinum mediated enrichment of ALDH + OCSCs in vivo*.*

## Discussion

Metabolic reprogramming is a distinctive characteristic of chemoresistant OC cells and likely occurs as a consequence of metabolic adaptation to the tumor microenvironment [16]. Metabolic reprogramming provides adenosine triphosphate [ATP] and precursors for macromolecular biosynthesis to meet the energy requirements for rapid proliferation and survival of tumor cells [53]. In addition to promoting tumorigenesis, alterations in cellular metabolism foster resistance to chemotherapy in OC and other cancer types [54–56]. The predominant mechanism of cytotoxicity of chemotherapeutic agents is inducing DNA damage and oxidative stress. Metabolic reprogramming enables tumor cells to adapt and manage pharmacological insults and oxidative stress [53]. The precise mechanism of how metabolic reprogramming promotes chemoresistance in OC is not understood. Several studies have established that ALDH + CSCs contribute to the development of chemoresistance in OC [7, 13, 14]. Our group and others have demonstrated that platinum treatment results in enrichment of ALDH + OCSCs [7, 13, 14, 52]. Persistence of these ALDH + OCSCs promotes tumor relapse and recurrence of chemoresistant disease contributing to high fatality rates among OC patients. Here, we demonstrate for the first time a role for OXPHOS and SIRT1 in the platinum-induced enrichment of ALDH + OCSCs.

There has been a long-standing debate about whether CSCs use glycolysis or OXPHOS for survival [57]. The metabolic pathway utilized by CSCs depends on which pathway provides a selective advantage in the tumor microenvironment. Here, we demonstrate that cisplatin treatment elevates expression of mitochondrial OXPHOS genes concurrently with an increase in percentage of ALDH + cells. Furthermore, OXPHOS inhibitors in combination with cisplatin or carboplatin block the enrichment of ALDH + cells in vitro and in vivo, suggesting that the platinum mediated increase in mitochondrial activity is required for the enrichment of ALDH + cells (Fig. 5F). The effect of platinum or mitochondrial complex I inhibitor IACS—010759 on other stem cell markers in OC like CD133, CD44 and others requires further investigation. However, our finding that IACS blocks cisplatin-induced spheroid formation suggests that IACS-010759 blocks platinum mediated enrichment of OCSCs. These findings agree with Viale et al. that pancreatic CSCs depend on OXPHOS for survival [19, 58]. It is likely that OXPHOS generates ROS to regulate metabolic plasticity of OCSCs, as recently suggested by Luo and Wicha [59].

Platinum resistant OC cells display elevated expression of the deacetylase SIRT1 [60, 61]. We demonstrate that SIRT1 levels and activity increase after platinum treatment. NAD + functions as a co-factor for several enzymes like ALDH and SIRT1 [21, 62], and the increase in SIRT1 activity and expression in response to cisplatin treatment may be due to platinum-induced increase in cellular NAD + levels [13, 14]. The dependency of the platinum mediated increase in ALDH + cells on SIRT1 may be due to enhanced regulation of the platinum-induced metabolic switch to oxidative metabolism and improved antioxidant defenses by SIRT1, both of which could promote survival of CSCs [45, 63]. SIRT1 supports mitochondrial function in muscle cells after exercise or starvation by regulating TFAM [64, 65], and we show that SIRT1 KD reduces the platinum-induced increase in *TFAM* expression. We hypothesize that the SIRT1-mediated increase in TFAM leads to an increase in OXPHOS gene expression leading to an increase in OXPHOS and ALDH activity (Fig. 5F). However, the precise mechanism of how SIRT1 contributes to the increase in ALDH + cells and regulates TFAM in response to cisplatin treatment requires further investigation.

Persistence of ALDH + OCSCs is a major obstacle in improving the survival rate of platinum resistant, recurrent ovarian cancer patients. Our group as well as others have demonstrated various therapeutic strategies to target this OCSC population thereby providing preclinical evidence to improve prognosis of OC patients with chemoresistant disease [7, 13, 52, 66]. Even though the lack of verification of our findings in patient derived tumor samples is a limitation of our study, this work has furthered our understanding of how metabolic alterations caused by platinum based chemotherapeutic agents can potentially be exploited to target ALDH + OCSCs. Although the involvement of OXPHOS in platinum resistance has been previously demonstrated [31, 67], our study has elucidated how platinum treatment reprograms the metabolism of OC cells resulting in enrichment of ALDH + OCSCs. Furthermore, our data demonstrated how this alteration in metabolism can be targeted using the small molecule inhibitor IACS-010759 to block the persistence of the platinum resistant ALDH + OCSC population. Our study supports further investigation into exploiting OXPHOS inhibitors like IACS-010759 to target the ALDH + OCSC population.

## Conclusions

The persistence of ALDH + cells after chemotherapy is a major cause of disease recurrence and relapse in OC [4]. We have demonstrated that cisplatin treatment of OC cells results in an increase in mitochondrial membrane potential. Our in vitro and in vivo data demonstrate that combined treatment with an OXPHOS inhibitor and platinum blocks the platinum-induced enrichment of ALDH + cells. Our findings reinforce the need for additional preclinical and clinical investigations aimed at exploiting OXPHOS inhibitors to delay tumor recurrence and improve survival in OC.

## Supplementary Information

**Additional file 1: Figure S1.** Mitochondrial DNA (mtDNA) content does not increase in response to cisplatin treatment. **A** Plots showing gates used to determine single cells of OVCAR5 cells untreated, OXPHOSi or 16 h cisplatin after JC-1 staining. **B** OVCAR5 cells were treated with IC50 dose of cisplatin for 16 h. DNA was isolated and qPCR was performed using two primer sets for different regions in the mitochondrial DNA (mtDNA) NADH dehydrogenase sub-unit 1 (ND1) and NADH dehydrogenase sub-unit 5 (ND5) relative to beta 2 microglobulin in the genomic DNA. Graph depicts mean ± SEM of mtDNA relative to nuclear DNA of N = 3 biological replicates, *P* values *< 0.05, **< 0.005, ***< 0.0005. **Figure S2.** Platinum treatment reduces HIF1α protein levels. **A** Western blot of HIF1α, c-Myc and Actin B in OVCAR3 cells treated with IC50 dose of cisplatin (15 µM) for 16 hours. **B** c-Myc expression by RT-qPCR in OVCAR3 cells untreated (U) or treated with IC50 dose (15 µM) cisplatin for 16 h. **Figure S3.** Treatment of OC cells with mitochondrial complex I inhibitor in combination with cisplatin blocks platinum-induced enrichment of ALDH+ cells. **A**, **B** Plots showing gates used to determine single cells in OVSAHO cells treated with cisplatin alone or in combination with DMSO or 1 µM IACS-010759 for 16 h after ALDELUOR assay. **C** Plots showing gates used to determine ALDH+ cells using DEAB controls for one biological replicate of OVSAHO cells. **Figure S4.** IACS treatment does not alter platinum-induced gene expression changes. Expression of OXPHOS genes (**A**) and c-Myc, HIF1α (**B**) in OVCAR5 cells treated with DMSO or IACS-010759 (1 μM) in combination with cisplatin (12 μM) for 16 h. **C** TFAM expression in OVCAR3 cells untreated or treated with IC50 dose of cisplatin (15 μM) for 16 h.

**Additional file 2: Table S1.** qRT-primer sequences.** Table S2.** Primers for mitochondrial DNA (mtDNA) content.

## Data Availability

All the data generated in the manuscript and materials used in the study are available upon reasonable request.

## Abbreviations

∆_Ψ__M_: Mitochondrial membrane potential
ALDH: Aldehyde dehydrogenase
ATM: Ataxia telangiectasia mutated
ATP5MF: ATP synthase membrane subunit F
COX5A: Cytochrome C oxidase subunit 5A
COX6A: Cytochrome C oxidase subunit 6A
CSCs: Cancer stem cells
DMSO: Dimethyl sulfoxide
HGSOC: High grade serous ovarian cancer
HIF1α: Hypoxia-inducible factor 1
HK1: Hexokinase 1
HK2: Hexokinase 2
KD: Knockdown
LDHA: Lactate dehydrogenase A
mtDNA: Mitochondrial DNA
NAD +: Nicotinamide adenine dinucleotide
NAMPT: Nicotinamide phosphoribosyltransferase
NDUFA11: NADH ubiquinone oxidoreductase subunit 11
NDUFS6: NADH ubiquinone oxidoreductase subunit 6
OC: Ovarian cancer
OCSCs: Ovarian cancer stem cells
OXPHOS: Oxidative phosphorylation
PDK1: Pyruvate dehydrogenase kinase
PKM2: Pyruvate kinase isozyme M2
RT-qPCR: Reverse transcriptase-quantitative polymerase chain reaction
ROS: Reactive oxygen species
shRNA: Short hairpin RNA
SIRT1: Sirtuin 1
TFAM: Mitochondrial transcription factor A
TIMM17A: Translocase of the inner mitochondrial membrane
UQCR10: Ubiquinol- cytochrome C reductase subunit X
UQCR11: Ubiquinol-cytochrome C reductase subunit XI
