# Supplementary material for: Platinum-induced mitochondrial OXPHOS contributes to cancer stem cell enrichment in ovarian cancer
Source: J Transl Med. 2022 May 31;20:246. doi: 10.1186/s12967-022-03447-y (PMC9153190; doi:10.1186/s12967-022-03447-y)
Supplement: Supplementary file 1 — Additional file 1: Figure S1. Mitochondrial DNA (mtDNA) content does not increase in response to cisplatin treatment. A Plots showing gates used to determine single cells of OVCAR5 cells untreated, OXPHOSi or 16 h cisplatin after JC-1 staining. B OVCAR5 cells were treated with IC50 dose of cisplatin for 16 h. DNA was isolated and qPCR was performed using two primer sets for different regions in the mitochondrial DNA (mtDNA) NADH dehydrogenase sub-unit 1 (ND1) and NADH dehydrogenase sub-unit 5 (ND5) relative to beta 2 microglobulin in the genomic DNA. Graph depicts mean ± SEM of mtDNA relative to nuclear DNA of N = 3 biological replicates, P values *< 0.05, **< 0.005, ***< 0.0005. Figure S2. Platinum treatment reduces HIF1α protein levels. A Western blot of HIF1α, c-Myc and Actin B in OVCAR3 cells treated with IC50 dose of cisplatin (15 µM) for 16 hours. B c-Myc expression by RT-qPCR in OVCAR3 cells untreated (U) or treated with IC50 dose (15 µM) cisplatin for 16 h. Figure S3. Treatment of OC cells with mitochondrial complex I inhibitor in combination with cisplatin blocks platinum-induced enrichment of ALDH+ cells. A, B Plots showing gates used to determine single cells in OVSAHO cells treated with cisplatin alone or in combination with DMSO or 1 µM IACS-010759 for 16 h after ALDELUOR assay. C Plots showing gates used to determine ALDH+ cells using DEAB controls for one biological replicate of OVSAHO cells. Figure S4. IACS treatment does not alter platinum-induced gene expression changes. Expression of OXPHOS genes (A) and c-Myc, HIF1α (B) in OVCAR5 cells treated with DMSO or IACS-010759 (1 μM) in combination with cisplatin (12 μM) for 16 h. C TFAM expression in OVCAR3 cells untreated or treated with IC50 dose of cisplatin (15 μM) for 16 h. [file 12967_2022_3447_MOESM1_ESM.docx]

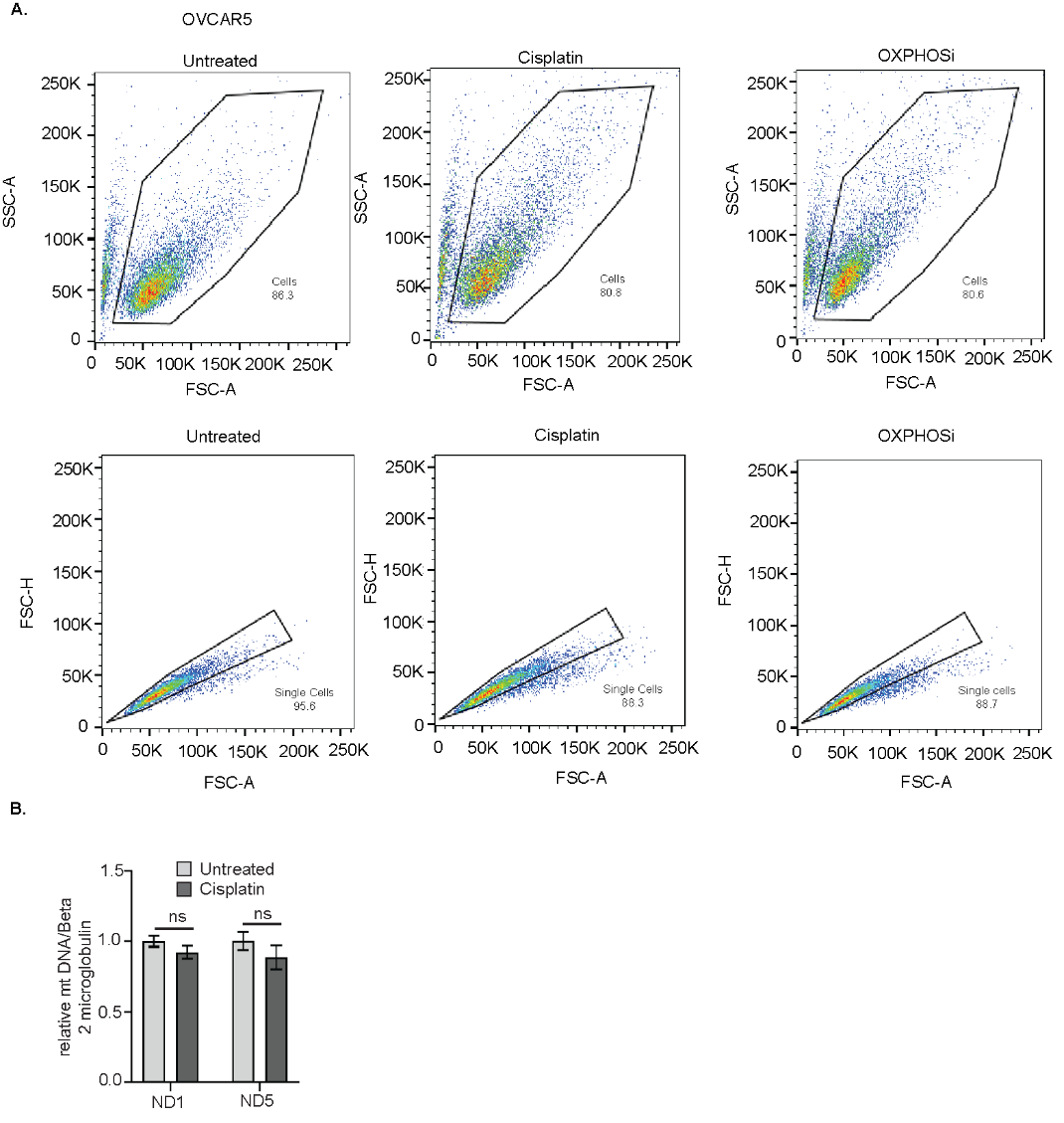


**Supplementary Figure S1. Mitochondrial DNA (mtDNA) content does not increase in response to cisplatin treatment. (A)** Plots showing gates used to determine single cells of OVCAR5 cells untreated, OXPHOSi or 16 hour cisplatin after JC-1 staining. **(B)** OVCAR5 cells were treated with IC50 dose of cisplatin for 16 hours. DNA was isolated and qPCR was performed using two primer sets for different regions in the mitochondrial DNA (mtDNA) NADH dehydrogenase sub-unit 1 (ND1) and NADH dehydrogenase sub-unit 5 (ND5) relative to beta 2 microglobulin in the genomic DNA. Graph depicts mean ± SEM of mtDNA relative to nuclear DNA of N=3 biological replicates, *P* values *<0.05, **<0.005, ***<0.0005.


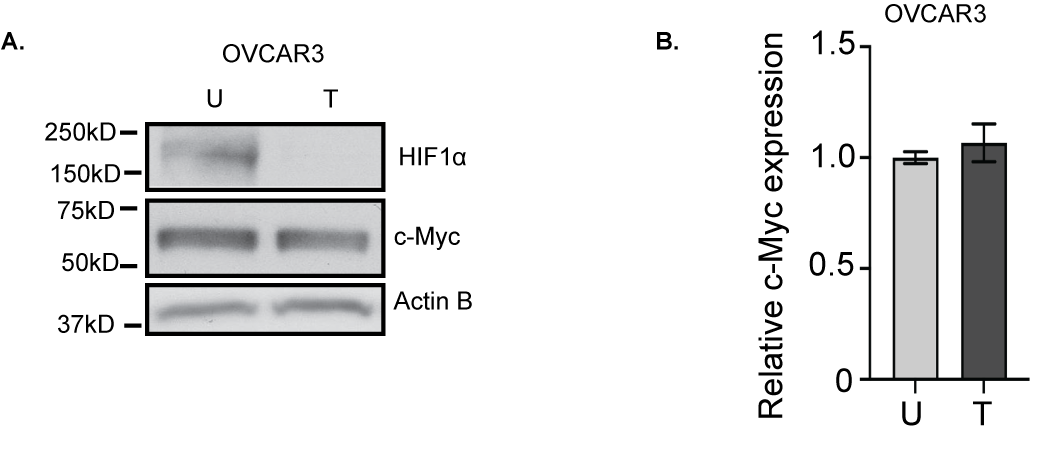


**Supplementary Figure S2. Platinum treatment reduces HIF1α protein levels. (A)** Western blot of HIF1α, c-Myc and Actin B in OVCAR3 cells treated with IC50 dose of cisplatin (15 μM) for 16 hours. **(B)** c-Myc expression by RT-qPCR in OVCAR3 cells untreated (U) or treated with IC50 dose (15 μM) cisplatin for 16 hours.


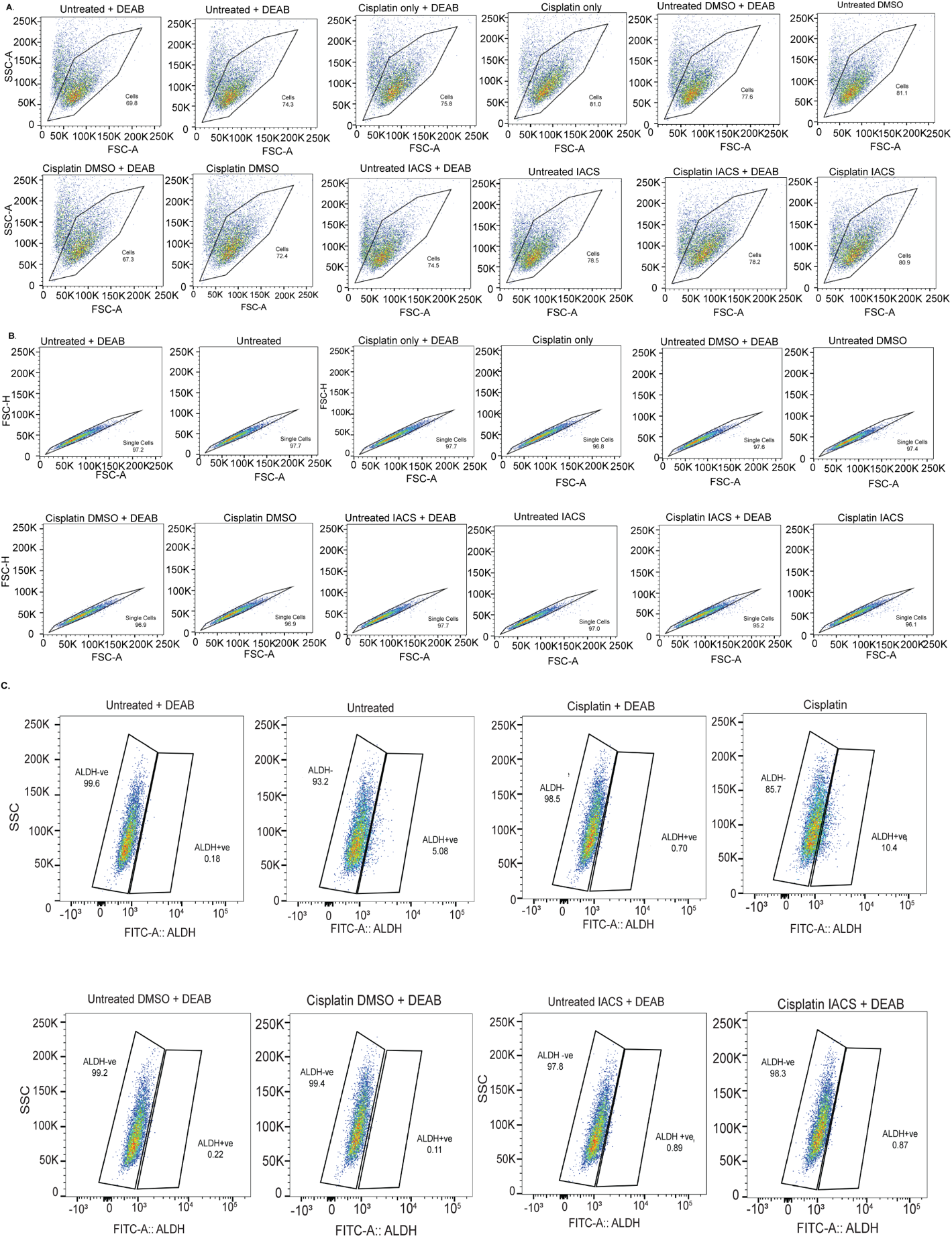


**Supplementary Figure S3. Treatment of OC cells with mitochondrial complex I inhibitor in combination with cisplatin blocks platinum-induced enrichment of ALDH+ cells. (A-B)** Plots showing gates used to determine single cells in OVSAHO cells treated with cisplatin alone or in combination with DMSO or 1 µM IACS-010759 for 16 hours after ALDELUOR assay. **(C)** Plots showing gates used to determine ALDH+ cells using DEAB controls for one biological replicate of OVSAHO cells


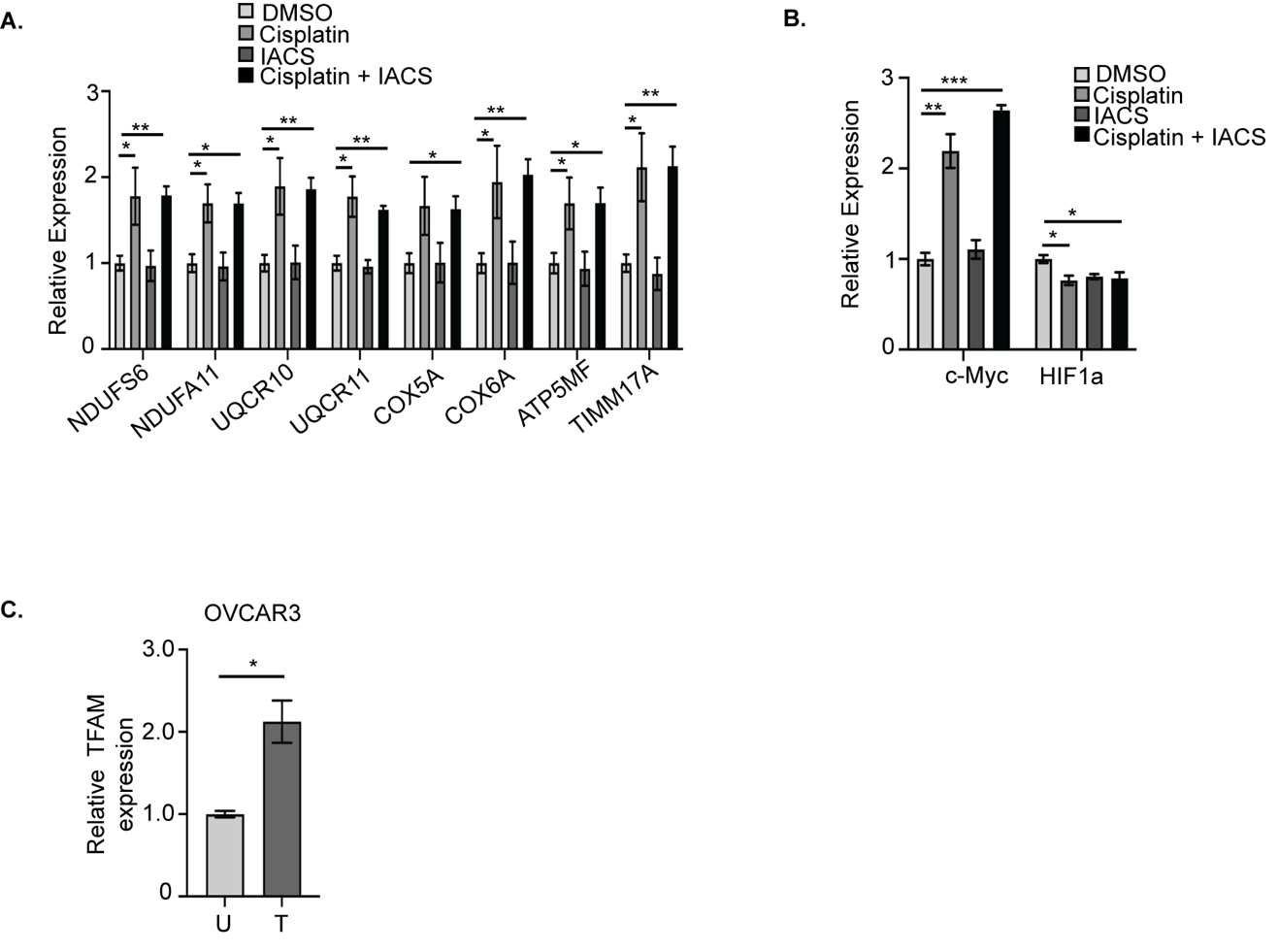


**Supplementary Figure S4**. **IACS treatment does not alter platinum-induced gene expression changes.** Expression of OXPHOS genes **(A)** and c-Myc, HIF1α **(B)** in OVCAR5 cells treated with DMSO or IACS-010759 (1 μM) in combination with cisplatin (12 μM) for 16 hours. **(C)** TFAM expression in OVCAR3 cells untreated or treated with IC50 dose of cisplatin (15 μM) for 16 hours.
