# Supplementary material for: Platinum-induced mitochondrial OXPHOS contributes to cancer stem cell enrichment in ovarian cancer
Source: J Transl Med. 2022 May 31;20:246. doi: 10.1186/s12967-022-03447-y (PMC9153190; doi:10.1186/s12967-022-03447-y)
Supplement: Supplementary file 2 — Additional file 2: Table S1. qRT-primer sequences. Table S2. Primers for mitochondrial DNA (mtDNA) content. [file 12967_2022_3447_MOESM2_ESM.docx]

**Table S1. qRT- Primer Sequences**

| Gene | Forward Primer | Reverse Primer | Primer  Efficiency |
| --- | --- | --- | --- |
| *NDUFS6* | TCTTGGCCACCCAAAAGTGT | CGGAAATGCTCACAGGATGC | 1.98 |
| *NDUFA11* | TGAACTACTTCCTCGGTGGC | CGCTATGCCAAAGTACACGC | 1.66 |
| *UQCR10* | TCGTGGGCGTCATGTTCTTC | GGGGGCCTCCAAGGAACTA | 1.67 |
| *UQCR11* | TCATCCTGAGGGTGCGACTC | GATCAGCCGCCAATCGGT | 1.69 |
| *COX5A* | TCATTGATGCTGCTTTGCGG | GTTCCTGGATGACATAGGGGT | 1.88 |
| *COX6A* | CATCTCCGCATCAGGACCAA | TCATCTTCGTAGCCAGTTGGA | 1.92 |
| *ATP5MF* | ACCAGGACTCCAAAATGGCG | CACTAGGACTGAAGTCCCGC | 1.78 |
| *TIMM17A* | GGTGGGGCCTTTACGATGG | GCCCTGGTTTTAATAGCTGTCA | 1.92 |
| *HIF1α* | TGAAGACATCGCGGGGAC | CTGGCTGCATCTCGAGACTTT | 2.01 |
| *c-Myc* | GGACCCGCTTCTCTGAAAGG | TAACGTTGAGGGGCATCGTC | 2.02 |
| *TFAM* | ACCAAAAAGACCTCGTTCAGC | TCAGAGTCAGACAGATTTTTCCAGT | 2 |
| *SIRT1* | TGACTGGACTCCAAGGCCACG | TCAGGTGGAGGTATTGTTTCCGGC | 2 |
| *Actin B* | GAAGCCGGCCTTGCACAT | AGCACAGAGCCTCGCCTTT | 2 |
| *HK1* | TGGCCTATTACTTCACGGAGC | AAATCCCGGGAGAGGCCATT | 2.03 |
| *HK2* | CGCCTCGGTTTCCCAACTCT | TCAGAGAGGCGCATGTGGTA | 2.12 |
| *LDHA* | CGCCGATTCCGGATCTCATT | AGCTGATCCTTTAGAGTTGCCA | 1.9 |
| *PDK1* | TCACCAGGACAGCCAATACA | ACCTCTGTTGGCATGGTGTT | 1.95 |
| *PKM2* | GGGCCATAATCGTCCTCACC | GACGAGCTGTCTGGGGATTC | 2.01 |

**Table S2. Primers for mitochondrial DNA (mtDNA) content**

| Gene | Forward Primer | Reverse Primer |
| --- | --- | --- |
| NADH dehydrogenase sub-unit 1 (ND1) | TTCTAATCGCAATGGCATTCCT | AAGGGTTGTAGTAGCCCGTAG |
| NADH dehydrogenase sub-unit 5 (ND5) | TTCATCCCTGTAGCATTGTTCG | GTTGGAATAGGTTGTTAGCGGTA |
| Beta 2 microglobulin | TGCTGTCTCCATGTTTGATGTATCT | TCTCTGCTCCCCACCTCTAAGT |
